# Characterization of Drugs with Good Glass Formers in Loaded-Mesoporous Silica and Its Theoretical Value Relevance with Mesopores Surface and Pore-Filling Capacity

**DOI:** 10.3390/ph15010093

**Published:** 2022-01-13

**Authors:** Arif Budiman, Diah Lia Aulifa

**Affiliations:** 1 Department of Pharmaceutics and Pharmaceutical Technology, Faculty of Pharmacy, Universitas Padjadjaran, Jl. Raya Bandung-Sumedang Km. 21, Bandung 45363, Indonesia; 2 Department of Pharmaceutical Analysis and Medicinal Chemistry, Faculty of Pharmacy, Universitas Padjadjaran, Jl. Raya Bandung-Sumedang Km. 21, Bandung 45363, Indonesia; diah.lia@unpad.ac.id

**Keywords:** mesoporous silica, good glass formers, maximum drug loading, monolayer covering surface of mesoporous, pore-filling capacity

## Abstract

The incorporation of a drug into mesoporous silica (MPS) is a promising strategy to stabilize its amorphous form. However, the drug within MPS has shown incomplete release, despite a supersaturated solution being generated. This indicates the determination of maximum drug loading in MPS below what is experimentally necessary to maximize the drug doses in the system. Therefore, this study aimed to characterize the drugs with good glass former loaded-mesoporous silica, determine the maximum drug loading, and compare its theoretical value relevance to monolayer covering the mesoporous (MCM) surface, as well as pore-filling capacity (PFC). Solvent evaporation and melt methods were used to load each drug into MPS. In addition, the glass transition of ritonavir (RTV) and cyclosporine A (CYP), as well as the melting peak of indomethacin (IDM) and saccharin (SAC) in mesoporous silica, were not discovered in the modulated differential scanning calorimetry (MDSC) curve, demonstrating that each drug was successfully incorporated into the mesopores. The amorphization of RTV-loaded MPS (RTV/MPS), CYP-loaded MPS (CYP/MPS), and IDM-loaded MPS (IDM/MPS) were confirmed as a halo pattern in powder X-ray diffraction measurements and a single glass transition event in the MDSC curve. Additionally, the good glass formers, nanoconfinement effect of MPS and silica surface interaction contributed to the amorphization of RTV, CYP and IDM within MPS. Meanwhile, the crystallization of SAC was observed in SAC-loaded MPS (SAC/MPS) due to its weak silica surface interaction and high recrystallization tendency. The maximum loading amount of RTV/MPS was experimentally close to the theoretical amount of MCM, showing monomolecular adsorption of RTV on the silica surface. On the other hand, the maximum loading amount of CYP/MPS and IDM/MPS was experimentally lower than the theoretical amount of MCM due to the lack of surface interaction. However, neither CYP or IDM occupied the entire silica surface, even though some drugs were adsorbed on the MPS surface. Moreover, the maximum loading amount of SAC/MPS was experimentally close to the theoretical amount of PFC, suggesting the multilayers of SAC within the MPS. Therefore, this study demonstrates that the characterization of drugs within MPS, such as molecular size and interaction of drug-silica surface, affects the loading efficiency of drugs within MPS that influence its relevance with the theoretical value of drugs.

## 1. Introduction

Over 70% of new drug candidates are poorly water-soluble, resulting in insufficient bioavailability via oral administration [1,2]. This means that developing a strategy to improve drug solubility is necessary to formulate poorly water-soluble drugs [3,4]. The amorphous system is a promising formulation strategy for increasing the solubility of drugs. Amorphous drugs have disordered structures and a higher Gibbs free energy than their crystalline counterparts [5]. Therefore, forming a supersaturated solution in the amorphous drug after being dispersed in water improves oral bioavailability [5,6]. However, the amorphous drug formulation is thermodynamically unstable and easily recrystallized during storage or its aqueous dispersion, thereby negating the advantages of enhanced solubility [7,8,9,10].

The incorporation of a drug into mesoporous silica (MPS) is a promising strategy to stabilize its amorphous form [11]. MPS is small enough carrier that is thermodynamically more favorable for the drug to remain in a disordered rather than crystalline state inside the pore [12]. Two responsible mechanisms explain the inhibition of drug crystallization, which include (i) the molecular interaction between functional groups of the drug molecules and the surface of MPS, such as hydrogen bonding and (ii) the nanoconfinement effect of MPS, leading to the suppression of nucleation and crystal growth of the drug as the pore size of MPS is smaller than the critical crystalline nuclei [13,14,15]. Considering the silica surface interaction, the large MPS surface area has an additional surface free energy and the drug adsorption in the amorphous state, which is thermodynamically favorable because of its lower free energy state than the crystalline drug [16,17]. When drug molecules occupy all the MPS surfaces, the excess drug has no direct contact with the MPS surface. Instead, it starts forming additional layers on the top of the initial drug monolayer [18,19]. Therefore, the crystallization from the excess amount of amorphous drug is being stabilized physically by the nanoconfinement effect of MPS and the surface area, and pore volume of MPS influence the loading capacity and crystallization of drugs in MPS [15,20].

To load a drug into the MPS, it needs to be temporarily mobilized. This has been achieved by melting the active pharmaceutical ingredients (API), allowing capillary forces to draw the melt into the pores [18,21], or by loading the API into the MPS from a solution. In this situation, the loading of drugs into MPS is generally conducted with three methods: temperature solid phase transformation (melt method), solvent immersion or incipient wetness impregnation [22]. The melt method is a solvent-free methodology based on the thermal transformation of drugs that is an efficient, alternative approach being used. However, this method is not considered a general approach, especially for drugs that cannot withstand melting without degradation. The high viscosity of the melted drug prevents successful drug loading. Meanwhile, the incipient wetness method, whereby the solution is dosed into dry carrier particles in MPS to fill the pores, is very beneficial due to the low energy demands of the process. The drugs should be dissolved at a high concentration in the solvent with low polarity to reach a high loading amount. Finally, the solvent evaporation method, in which the carrier particles are dispersed in the API solution, followed by complete evaporation of the solvent [23,24,25], is the most general method and provides high drug loadings. However, the possibility of recrystallization on the surface of MPS needs to be considered in the case of exceeding the pore capacity. Therefore, choosing a loading method is crucial for high drug loading into MPS [12].

Even though many studies have reported drug loading into various silica carriers under specific conditions [21,22,26,27,28,29], the determination of the maximum drug loading amount into MPS is not clearly understood, particularly for the amorphous drugs categorized in class III, based on Taylor’s classification, which has good glass formers that neither crystallize upon cooling nor reheating [15,29]. It is necessary to determine maximum drug loading experimentally to maximize its effect because drugs in MPS have shown incomplete release, although a supersaturated solution has been shown to be generated. To overcome the aforementioned inconsistencies concerning the drug loading capacity determinations, differential scanning calorimetry (DSC) with good glass-forming ability has been used in this study to determine the maximum loading of an amorphous drug. The amorphous nifedipine, which was loaded into a nanoconfinement system, exhibited a lower glass transition (*T*_g_) than the bulk amorphous, indicating a higher molecular mobility amorphous nifedipine in the nanoconfinement system [26]. However, other studies have reported that the molecular mobility of drugs loaded with small enough pores is significantly lower than in bulk. This indicates the *T*_g_ will be higher than the bulk amorphous [27,28]. On the other hand, the glass transition of the drug, which adsorbed monomolecularly on the silica surface, was not detected, as the monolayer of the drug on the silica surface does not contribute to the *T*_g_ signal in the DSC. The presence of *T*_g_ has been attributed to an excess amorphous phase in MPS or unloaded drug in MPS [15,29,30,31]. Therefore, an investigation from *T*_g_ of drug-loaded MPS should be necessary to determine the maximum drug loading.

This study systematically characterized the amorphous drug with good glass formers that neither crystallize upon cooling nor upon reheating (class III) and determined maximum drug loading below the experimentally known amounts. Moreover, we also compared the maximum loading amount of drug with theoretical value referred to monolayer covering surface of mesoporous (MCM) and pore-filling capacity (PFC). The drugs were selected to cover those poorly soluble categorized in class III with various molecular weights. Ritonavir (RTV), cyclosporine A (CYP) and indomethacin (IDM) were used as models of poorly water-soluble drugs categorized in class III [32]. Meanwhile, saccharin (SAC), with a high recrystallization tendency (class I) [31], was also evaluated as a comparison study. The solvent evaporation method and melt method were adopted for loading the drug into MPS. In addition, the characterization of amorphous drugs and the maximum drug loading was determined by modulated differential scanning calorimetry (MDSC) analysis. In contrast, the amorphization of the drug was evaluated using X-ray powder diffraction (XRPD) analysis.

## 2. Results

MPS used in this study is ordered mesoporous silica and has a porous texture in accordance with the mesoporous silica materials. According to the IUPAC classification, MPS shows a typically irreversible type IV isotherm. The pore volume, specific surface area and pore diameter of MPS_1_ were 0.92 cm^3^/g, 820 nm^2^/g and 8 nm, respectively, while for MPS_2_ were 1.2 cm^3^/g, 550 nm^2^/g and 6 nm, respectively. The MPS was characterized by Fourier-transform infrared spectroscopy (FT-IR) measurement, as shown in Figure S1. Previous studies reported that MPS possesses a characteristic signal at around 3749 cm^−1^ assigned to the stretching vibrations of isolated (i.e., non-hydrogen bonded) silanol groups [30]. MS_1_ and MS_2_ exhibited a characteristic signal at 3750 cm^−1^, attributed to the stretching vibrations of isolated (i.e., non-hydrogen-bonded) silanol groups. This indicated that OH groups were observed on the surface of MPS_1_ and MPS_2_. To confirm the presence of OH groups on the silica surface, solid-state ^29^Si NMR measurement was also performed as shown in Figure S2. The Q2 and Q3, which was attributed to the silanol groups, were observed in both MS_1_ and MS_2_. This indicated that it could only be OH groups on the surface of MPS, which was in agreement with the FT-IR measurement.

### 2.1. Characterization of RTV-Loaded MPS_1_ (RTV/MPS_1_)

**Figure 1:**
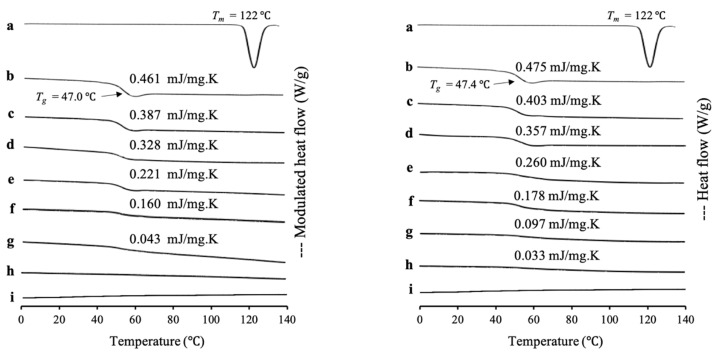
(**Left**) MDSC curve and (right) DSC curve of (a) RTV crystal, (b) (left) RTV EVPs, (**right**) RTV Ms, RTV/MPS_1_ = (c) 8:2, (d) 7:3, (e) 6:4, (f) 5:5, (g) 4:6, (h) 3:7 and (i) MPS_1_. (**Left**) RTV/MPS_1_ prepared by solvent evaporation and (**right**) melt method. Nonreversible signal is shown in Figure S3.

RTV/MPS_1_ prepared by the solvent evaporation method and melt method with weight ratios was evaluated by MDSC measurement, as shown in Figure 1. The RTV crystal showed a melting peak at 122 °C, while the amorphous RTV prepared by solvent evaporation (RTV EVPs) and melt method (RTV Ms) showed a glass transition event at 47.0 °C and 47.4 °C, respectively, and did not show a melting peak. This indicated an amorphous drug, which has good glass formers that does not crystallize even upon reheating. The heat capacity changes (Δ*Cp*) of *T*_g_ of both RTV EVPs and RTV Ms decreased with a decrease in RTV concentration in MPS_1_, while the *T*_g_ remained constant. In RTV/MPS_1_ prepared by a solvent evaporation method, the glass transition event for RTV EVPs was observed in the weight ratio above 4:6. However, in the weight ratio of 3:7, the glass transition of RTV EVPs was not detected in the MDSC curve. The absence of a glass transition event of RTV EVPs was attributed to the monomolecular adsorption of RTV on the silica surface of MPS_1_ [29,30]. The remained glass transition event for RTV EVPs at the weight ratio above 4:6 showed that some RTV existed as amorphous RTV outside the pores of MPS_1_ [21,33].

Although the *T*_g_ decreased with a decrease in RTV concentration in MPS_1_, the glass transition event for RTV Ms was still observed in the weight ratio 3:7 of RTV/MPS_1_ prepared by melt method, indicating some of RTV existed as amorphous RTV outside of the pore of MPS_1_. Furthermore, the amount of RTV-loaded MPS_1_ by the melt method was lower than the solvent evaporation method. This may be due to the high viscosity of molten RTV, which may impede the flow of liquid into the pores and lead to the failure of liquid RTV intrusion into the pores of MPS_1_ [21].

**Figure 2:**
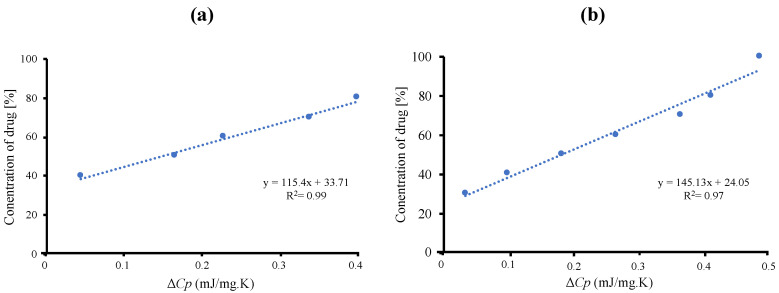
Plots of the concentration of RTV against Δ*Cp* of amorphous RTV calculated from the DSC curves. (**a**) RTV/ MPS_1_ prepared by solvent evaporation and (**b**) melt method.

The maximum amount of RTV-loaded MPS_1_ is determined quantitatively by Δ*Cp* values of amorphous RTV on the MDSC curves. The concentration of amorphous RTV was plotted as a function of the Δ*Cp*, as shown in Figure 2. The fitted lines for RTV/MPS_1_ prepared by solvent evaporation method showed good linearity with correlation coefficients of 0.98, while the correlation coefficients of RTV/MPS_1_ prepared by melt method were 0.97. The y-intercept value represents the maximum amount of RTV-loaded MPS_1_. The maximum RTV EVPs and Ms-loaded MPS were 33.71% (*w*/*w*) and 24.05% (*w*/*w*), respectively. This showed that the solvent evaporation method is more efficient in the loading RTV into MPS_1_ compared to the melt method.

To confirm the relevance of loading amount with theoretical value, the theoretical calculation of RTV-loaded MPS_1_, referred to as theoretical monolayer coverage of drug within MPS_1_, as well as pore-filling capacity, were calculated using Equations (1) and (2), where the maximum projected surface area (S_drug_) of RTV is 276.64 Å^2^ [34]. At the same time, the powder densities of amorphous RTV are 1.239 cm^3^/g [35]. The result showed that the theoretical amount of RTV required for a monolayer coverage of MPS_1_ was 34.5% (*w*/*w*). Meanwhile, the theoretical amount of RTV needed to fill the pores of MPS_1_ was 53.3% (*w*/*w*). Thus, the maximum loading amount of RTV/MPS_1_ prepared by the solvent evaporation method was very close to the theoretical calculation of MCM. Therefore, it was assumed that after being incorporated into MPS_1_, RTV was monomolecularly adsorbed on the silica surface of MPS_1_. In addition, the theoretical calculation of PFC was almost similar to RTV/MPS_1_ with a weight ratio of 5:5. In this ratio, the *T*_g_ was observed, which attributed to some of RTV existing as amorphous outside of the pore of MPS_1_. Therefore, to estimate the maximum loading amount of RTV into MPS_1_, the theoretical calculation of MCM was suggested to be used due to close agreement experimentally with the maximum loading amount.

**Figure 3:**
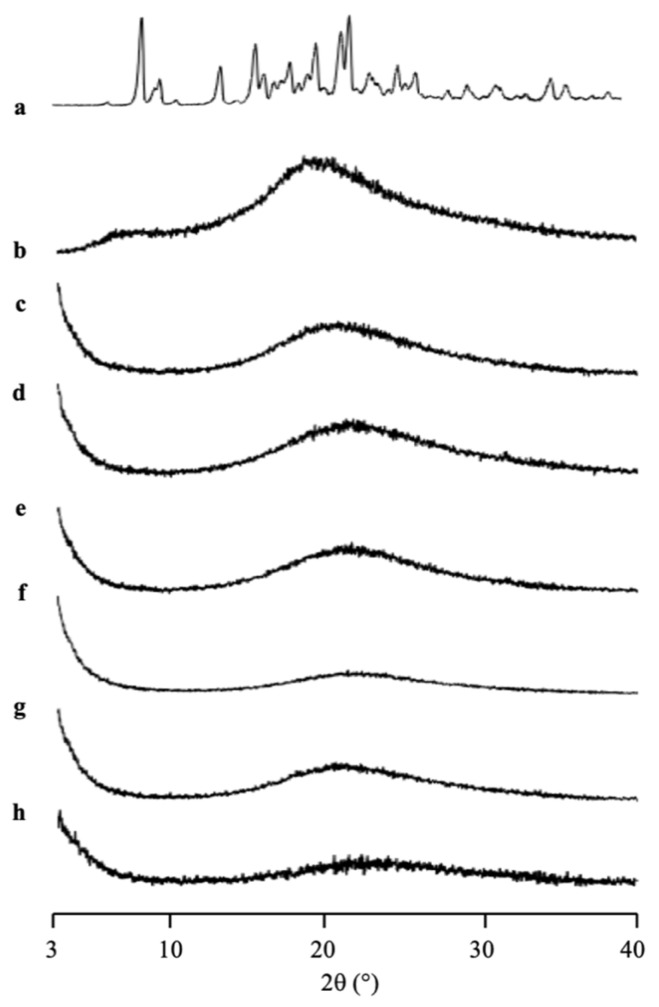
The PXRD patterns of (a) RTV crystal, (b) RTV EVPs, RTV/MPS = (c) 8:2, (d) 7:3, (e) 6:4, (f) 5:5, (g) 3:7 and (h) MPS_1_.

The amorphization of RTV/MPS_1_ with various weight ratios prepared by solvent evaporation method was evaluated by PXRD measurement (Figure 3). The RTV crystal showed characteristic diffraction peaks in the PXRD patterns. In contrast, the amorphous RTV prepared by solvent evaporation showed a halo pattern without any diffraction peaks. Furthermore, the characteristic diffraction peaks of RTV crystal were also not observed in all weight ratios of RTV/MPS_1_. This implies that the RTV crystal was amorphized by solvent evaporation. Moreover, RTV was categorized into class III based on Taylor’s classification, which has good glass formers that neither crystallize upon cooling nor upon reheating [15,31,32]. The amorphous RTV was stable, while some RTV was outside of MPS_1_ for the higher weight ratios of RTV/MPS_1_.

### 2.2. Characterization of CYP-Loaded MPS_1_ (CYP/MPS_1_)

**Figure 4:**
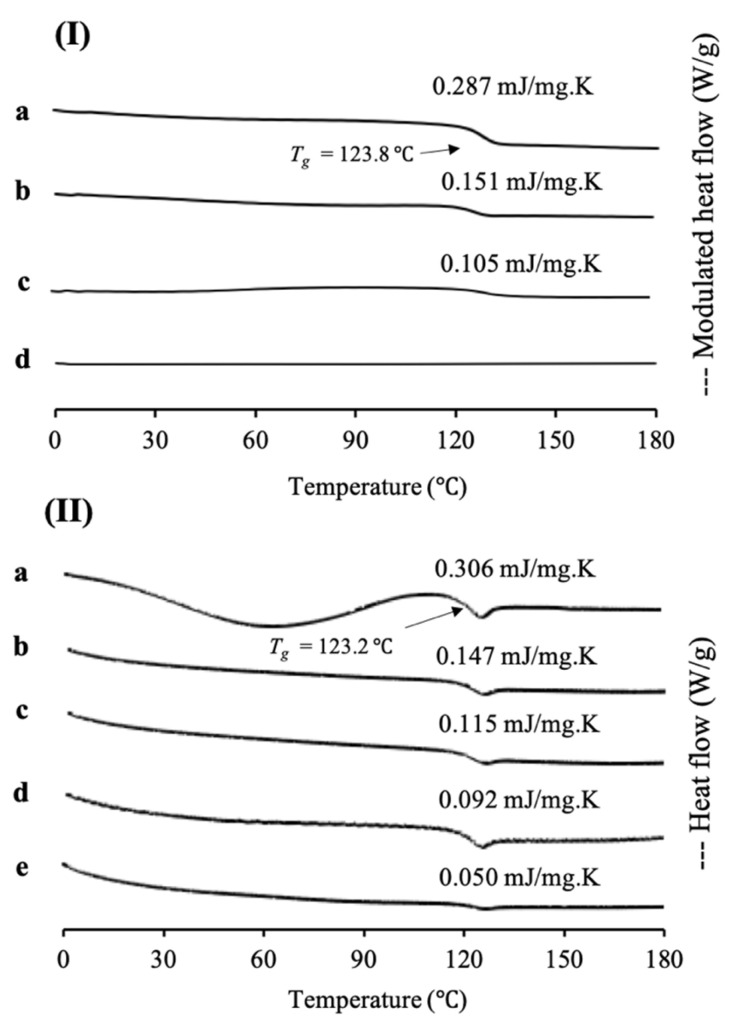
(**I**) MDSC curve and (**II**) DSC curve of (a) CYP amorphous state, CYP/MPS_1_ = (b) 7:3, (c) 5:5, (d) 3:7 and (e) 2:8. (**I**) CYP/MPS_1_ prepared by solvent evaporation and (**II**) melt method. Nonreversible signal is shown in Figure S4.

In this study, cyclosporine A was used as a model of a poorly water-soluble drug, as its sample was already in an amorphous state, according to class III of Taylor’s classification and has a high molecular weight. MDSC and XRPD analyses were conducted to characterize CYP/MPS_1_ with various weight ratios. MDSC measurements were also performed to determine the loading amount of CYP into MPS_1_ (Figure 4). The MDSC curve of CYP EVPs and CYP amorphous states exhibited an endothermic peak at 123.8 °C and 123.2 °C, respectively, which attributed to their glass transition event and no thermal event occurred, showing that there was no recrystallization of CYP upon reheating. Similar to RTV/MPS_1_, the ΔCp of the glass transition event from CYP also decreased with a decrease in the CYP concentration either in CYP/MPS_1_ prepared by a solvent evaporation or melt method. In CYP/MPS_1_ prepared by a solvent evaporation method, the glass transition event for CYP was observed in the weight ratios of 7:3 and 5:5, indicating some of CYP existed in an amorphous state outside of the mesopores. In contrast, the glass transition of CYP/MPS_1_ = 3:7 was not detected in the MDSC curve. The absence of a glass transition event indicated that CYP was monomolecularly adsorbed on the silica surface of MPS_1_, which agreed with RTV/MPS_1_, as well as other previous studies [24]. Thus, the loading of 30% CYP could be almost the maximum value to be loaded MPS_1_. On the other hand, the glass transition event for CYP was still observed even in the weight ratio 2:8 of CYP/MPS_1_ prepared by the melt method, indicating that some CYP was still outside of MPS_1_. The amount of CYP-loaded MPS_1_ by the melt method was lower than that by the solvent evaporation method. Similar to RTV/MPS_1_, the high viscosity of molten CYP led to the failure of liquid RTV intrusion into the mesopore [21].

The maximum amount of CYP-loaded MPS_1_ was also determined quantitatively by plotting between the concentration of the CYP amorphous state and its Δ*Cp* values (data not shown). The maximum amount of CYP/MPS_1_ prepared by solvent evaporation and melt method was 26.9% and 8.1%, respectively, with a linear coefficient of determination (R^2^) value of 0.98 for the solvent evaporation method and 0.95 for the solvent evaporation melt method. Similar to RTV/MPS_1_, these results showed that the solvent evaporation method is more efficient in the loading CYP into MPS_1_ compared to the melt method. A previous study reported that the maximum projected S_drug_ of CYP is 279 Å^2^ [36], while the powder densities of CYP are 1.159 cm^3^/g [37]. Thus, the theoretical amount of CYP required for a monolayer coverage of MPS_1_ was 59.3% (*w*/*w*), while the theoretical amount of CYP needed to fill the pores of MPS_1_ was 51.6% (*w*/*w*). These values were significantly different compared to the maximum amount of CYP/MPS_1_ obtained experimentally. Moreover, it was assumed that CYP did not completely occupy the silica surface of MPS_1_ due to the weak interaction between CYP and the silica surface of MPS_1_. Thus, in the CYP/MPS_1_ = 5:5, the *T*_g_ was observed due to some CYP existing in an amorphous state outside of the mesopores. However, this weight ratio was lower than the theoretical amount of CYP required for a monolayer coverage of MPS_1_. Further investigation is needed to confirm the interaction between CYP and the silica surface of MPS_1_. Based on this result, the theoretical calculation of MCM and PFC is not adequate to estimate the maximum loading amount of CYP into MPS_1_.

**Figure 5:**
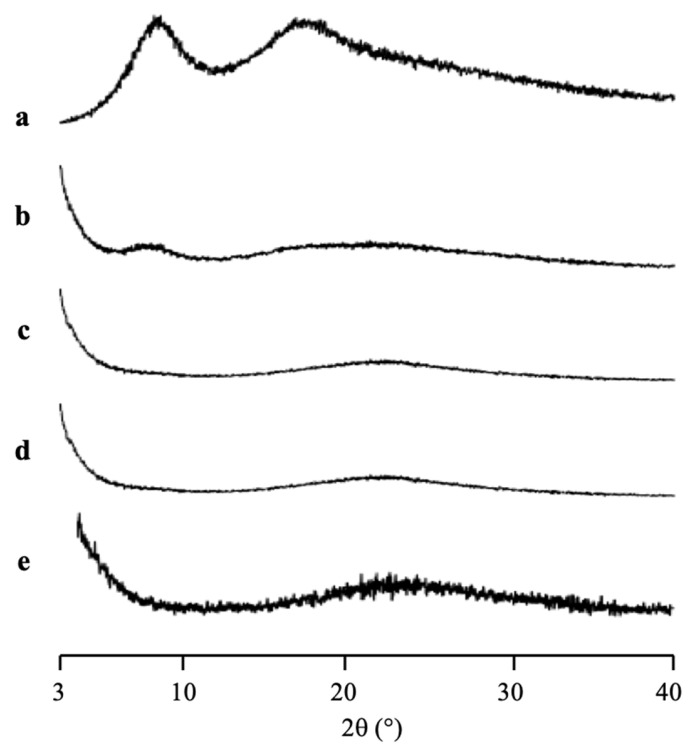
The PXRD patterns of (a) CYP amorphous state, CYP/MPS_1_ = (b) 7:3, (c) 5:5, (d) 3:7 and (e) MPS_1_.

The PXRD patterns of CYP/MPS_1_ with various weight ratios prepared by solvent evaporation method are shown in Figure 5. The CYP amorphous state and all ratios of CYP/MPS_1_ showed a halo pattern without any diffraction peaks, indicating the amorphization of RTV/MPS_1_. Similar to RTV, this result was representative of the CYP amorphous state being stable. However, some CYP was outside of MPS_1,_ specifically for the higher weight ratios of CYP/MPS_1_, due to the good glass formers that neither crystallize upon cooling nor upon reheating.

### 2.3. Characterization of IDM-Loaded MPS_2_ (IDM/MPS_2_)

**Figure 6:**
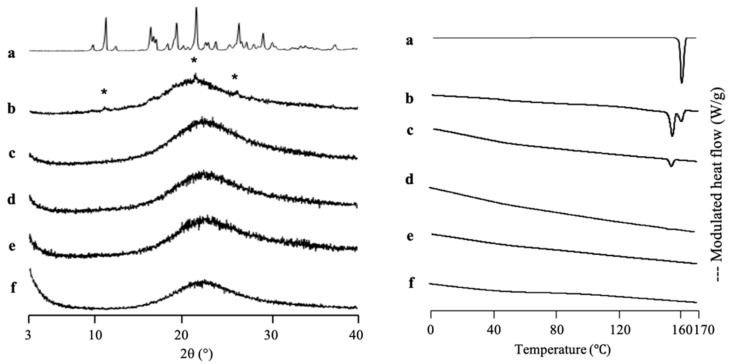
(**Left**) The PXRD patterns and (**right**) MDSC curve of (a) *γ*-IDM crystal, IDM/MPS_2_ = (b) 4:6, (c) 3:7, (d) 2:8, (e) 1.5:8.5 and (f) MPS_2_. Nonreversible signal is shown in Figure S5. * Characteristic peaks of IDM crystal.

IDM was used as a model for the poorly water-soluble drug due to its class III and low molecular weight. Furthermore, it was loaded into another MPS (MPS_2_) as a comparison study. The solvent evaporation method was used for IDM loading into MPS_2_, due to the greater efficiency based on two previous drugs loading into MPS_1_. The thermal analysis and the amount of amorphous IDM in the MPS_2_ mesopores were also investigated by MDSC measurement (Figure 6). The DSC curve of the IDM crystal (*γ*-IDM) showed an endothermic peak at 161 °C, which corresponds to its melting point. On the other hand, MPS_2_ did not show glass transition events and melting peaks in MDSC curves. The heat of fusion of IDM decreases with a decrease in IDM concentration. In the weight ratios of 3:7 and 4:6, the melting peak of IDM crystal was observed, showing that some of IDMs were possibly on the outer surface of the MPS pores. The IDM/MPS_2_ = 4:6 exhibited crystallization at 101 °C, which attributed to *α*-IDM and melting of *γ*-IDM, indicating the existence of *α*-IDM crystals that were obtained after being prepared by the solvent evaporation method. In contrast, the melting peak almost disappeared in IDM/MPS_2_ at a ratio of 2:8, showing that nearly all of IDM was successfully loaded into MPS_2_. Thus, the loading of 20% IDM could be almost the maximum value to be loaded into MPS_2_.

For IDM/MPS_2_, we did not determine the maximum loading amount of IDM into MPS_2_ by plotting between the heat of fusion of IDM and IDM concentration. The presence of two endothermic peaks could results in inaccurate data in determining the maximum loading amount of IDM into MPS_2_. Thus, we predicted the maximum loading amount of IDM into MPS_2_ based on the absence of its melting peak. Next, the theoretical amount of IDM, referred to as theoretical MCM and PFC, was calculated. A previous study reported that the maximum projected S_drug_ of IDM is 122 Å^2^ [38], while the powder densities of IDM are 1.32 cm^3^/g [39]. Thus, the theoretical amount of IDM required for a monolayer coverage of MPS_2_ was 26.75% (*w*/*w*), while the theoretical amount of IDM needed to fill the pores of MPS_2_ was 61.73% (*w*/*w*). The actual ratio of drugs within nanoconfinement is generally lower than the theoretical value [1,26]. Moreover, McCarthy et al. (2020) reported that a crystalline peak characteristic of *γ*-IDM polymorph was observed at the level of 75% or above from the theoretical amount of IDM referred to as theoretical MCM within SBA-15 pores, indicating that some drugs may be outside of the pores [38]. Thus, the incorporation of 20% IDM into MPS_2_ in the experiment was quite reasonable, as this amount was lower (almost equal to 75%) than the theoretical value (26.75%).

A PXRD measurement was also conducted to investigate the incorporation of IDM into MPS_2_ mesopores (Figure 6). The *γ*-IDM crystal showed characteristic peaks of IDM in the PXRD pattern. The peak positions of IDM crystals were consistent with those in a previous report [40,41]. The diffraction patterns of IDM/MPS_2_ = 4:6 demonstrated peaks corresponding to IDM crystals, indicating some IDM existing as crystal outside of mesopore. Meanwhile, IDM/MPS_2_ = 2:8 showed PXRD halo patterns demonstrating that MPS_2_ entraps almost all IDM. In addition, the incorporation of IDM into the pores of MPS_2_ changes its molecular state from crystalline to amorphous.

### 2.4. Characterization of SAC-Loaded MPS_1_ (SAC/MPS_1_)

**Figure 7:**
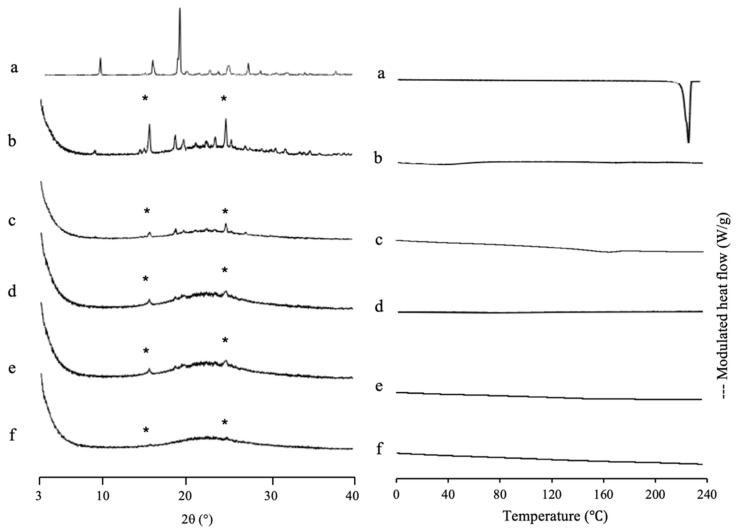
(**Left**) PXRD pattern and (**right**) MDSC curve of (a) SAC crystal, evaporated sample of SAC/MPS_1_ = (b) 4:6, (c) 3:7, (d) 2:8, (e) 1.5:8.5 and (f) 1:9. Nonreversible signal is shown in Figure S6. * Characteristic peaks of SAC crystal.

SAC, which has a high recrystallization tendency (class I), was used as a comparison study. Figure 7 shows the MDSC curve of SAC/MPS_1_ with various weight ratios. The DSC curve of SAC showed an endothermic peak at 228 °C, which corresponds to its melting point. Meanwhile, the *T*_g_ of SAC prepared by the solvent evaporation method was not detected and just showed its endothermic peak (data not shown). This could be due to the high crystallization tendencies of SAC categorized into class I of Taylor’s classification. Interestingly, the melting peak of SAC crystal was not observed even in NIC/MPS_1_ = 4:6. The absence of a melting peak of SAC indicated that almost all SAC was successfully loaded into MPS_1_ [42]. Next, the theoretical amount of SAC referred to MCM and PFC was calculated. The maximum projected S_drug_ of SAC is 71.6 Å^2^ [43], while the powder densities of SAC crystal are 0.828 cm^3^/g [44]. Thus, the theoretical amount of SAC required for a monolayer coverage of MPS_1_ was 34.83% (*w*/*w*), while the theoretical amount of SAC needed to fill the pores of MPS_1_ was 43.24% (*w*/*w*). The result of the maximum loading amount of SAC/MPS_1_ was very close to the theoretical calculation of PFC. Thus, it was assumed that after being incorporated into MPS_1_, SAC was both on the silica surface of MPS_1_ and in the center of the pore. Moreover, to estimate the maximum loading amount of SAC into MPS_1_, the theoretical calculation of PFC is suggested to be used, due to close agreement with the maximum loading amount experimentally.

The PXRD pattern of SAC/MPS_1_ with various weight ratios is also shown in Figure 7. The SAC crystal showed characteristic peaks of crystalline SAC in the PXRD pattern. The diffraction peak characteristic of crystalline SAC was also observed in the evaporated SAC samples due to the high recrystallization tendency (data not shown). Interestingly, the SAC/MPS_2_ system showed diffraction peaks characteristic of the PXRD patterns in the weight ratio of 4:6 and even 1:9, although all SAC was incorporated into MPS_1_ based on MDSC measurement. The crystallization of SAC was seen both within and outside MPS_1_. The crystallization of SAC within MPS_1_ was suggested to occur because there was no or weak interaction between SAC and the silica surface of MPS_2_. Moreover, the difference between the SAC molecule and the pore size of MPS_1_ is extremely high. Thus, the critical nucleus size of SAC was formed within MPS_1_, which led to the recrystallization of SAC [31].

**Figure 8:**
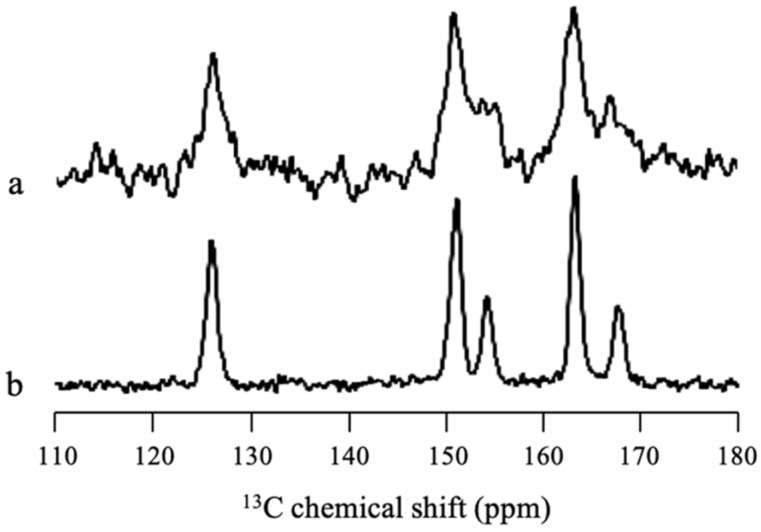
^13^C CP/MAS NMR spectra (υ = 5 kHz) of (a) SAC/MPS_1_ = 2:8 and (b) SAC crystal.

Solid-state NMR measurement was performed to confirm whether there was some SAC amorphization in MPS_1_ or all SAC in MPS_1_ was in the crystalline state. Figure 8 revealed that some peaks of SAC/MPS_2_ = 2:8 were broadener compared to SAC crystal, reflecting the wide distribution of chemical shifts of SAC peaks. This indicated that the amorphization of SAC in MPS_1_ occurred due to interaction between SAC and the silica surface of MPS_1_, although most of SAC within MPS_1_ was in the crystalline state.

## 3. Discussion

The drug in an amorphous state has excess free energy compared with the crystalline state. Therefore, no energy is required to break the crystal lattice structure, which positively affects the dissolution rate and solubility [45]. The incorporation of the drug into MPS stabilizes the amorphous form of the drug through the nanoconfinement effect of MPS and molecular interaction between functional groups of the drug and the silica surface of MPS. This study systematically elucidated the characterization of loading amorphous drugs with good glass formers (class III), the experimental determination of maximum drug loading and its comparison with theoretical value referred to MCM and pore PFC.

**Figure 9:**
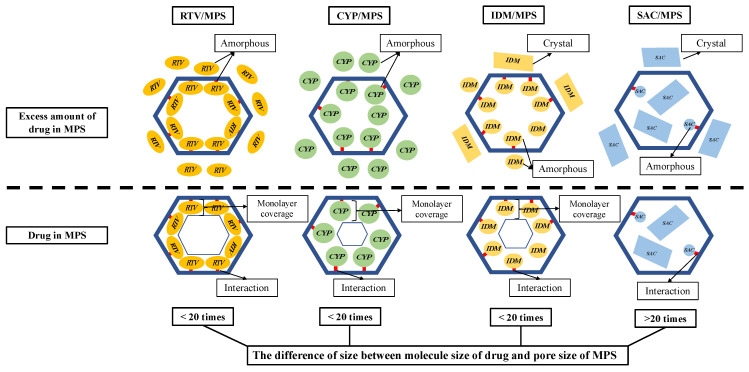
Schematic illustration of RTV/MPS, CYP/MPS, IDM/MPS and SAC/MPS.

A speculated mechanism of each drug within MPS is discussed in this study (Figure 9). In the RTV/MPS_1_ system, RTV was amorphized by the solvent evaporation method. In MDSC measurement, the RTV was stable in an amorphous state even after heating, either in RTV alone or in the RTV/MPS_1_ system. This could be due to RTV being a drug with a low recrystallization tendency (class III) and a good glass former that neither crystallizes upon cooling nor upon reheating. Moreover, for RTV/MPS_1_, the nanoconfinement effect from MPS could further stabilize the amorphous state of RTV, due to the different sizes between the molecule size of RTV and the pore size of MPS_1_, which was not more than 20 times. The size of RTV was 18.2 Å × 15.2 Å, while the pore size of MPS_1_ was 80 Å. Previous studies have reported that drug recrystallization occurred within the MPS if the pore size was 20 times larger than the size molecules of the drug [31,46,47]. Therefore, MPS could suppress the critical nucleus size of RTV. The maximum loading amount of RTV/ MPS_1_ prepared by the solvent evaporation method was very close to the theoretical calculation of MCM, indicating that RTV was monomolecularly adsorbed on the surface of MPS_1_. A previous study reported that the hydrogen bonding between the C=O of RTV and the Si-OH of MPS was observed [48]. This strong interaction contributed to the monomolecular adsorption of RTV on the silica surface of MPS_1_. In contrast, the theoretical value of PFC was significantly higher than the experimental maximum drug loading; this could be due to the relatively small average pore size of MPS_1_ for RTV, resulting in a spatial limitation. Moreover, some pores may be too narrow to accommodate the molecules to be multilayers in the entire MPS_1_. Therefore, RTV was only able to cover the silica surface of MPS_1_.

Due to its low recrystallization tendency, the CYP/MPS1 was also stable in an amorphous state even after heating in all weight ratios. Similar to RTV, the difference in size between the molecule size of CYP and the pore size of MPS_1_ was not more than 20 times. Thus, recrystallization of CYP was efficiently inhibited by the nanoconfinement effect of MPS_1_ and its theoretical amount, referred to as either MCM or PFC, was about 50%. However, some CYP existed as an amorphous state outside of the mesopore in the CYP/MPS_1_ = 5:5. The interaction of the CYP-silica surface was not stronger than the RTV-silica surface. Thus, CYP did not occupy the entire silica surface, as some CYP was carried away from mesopores by the solvent in the drying process, leading to a decrease in the loading amount of CYP. Moreover, the space of MPS_1_ was not enough for CYP, which has a high molecular weight, to form multilayers in the entire MPS_1_. However, further investigation is still needed to confirm the interaction between CYP and the silica surface of MPS_1_.

The presence of *T*_g_ in the MPS system was possibly attributed to either loaded or unloaded drugs in mesoporous silica. Previous study reported a similar *T*_g_ of the drug was observed when the amount of drug incorporated into mesoporous silica was higher than the maximum loading of the drug [21,30]. On the other hand, when the amount of drug was lower than the maximum loading of the drug, the *T*_g_ of the drug was not observed. Other studies reported that the *T*_g_ of the drug in the mesoporous silica would be changed due to the different mobility of amorphous drugs [26,27]. The *T*_g_ event of the RTV amorphous state in the weight ratio of RTV/MPS_1_ of above 4:6 and CYP amorphous state in the CYP/MPS_1_ = 5:5 was almost similar with the RTV amorphous and CYP amorphous states, respectively. Thus, it was assumed that a glass transition event in the weight ratio of RTV/MPS_1_ and CYP/MPS_1_ was derived from excess RTV and CYP amorphous states, which were not incorporated into the mesopores of MPS.

In the IDM/MPS2 system, although it belongs to class III, IDM crystallized after heating both in IDM alone and in RTV/MPS2. This could be due to the molecular size of IDM being smaller compared to RTV and CYP; thus, the molecular mobility would be higher, which leads to critical nucleus formation and subsequent recrystallization. The IDM was amorphized in MPS_2_ by the nanoconfinement effect of MPS_2_. Similar to RTV and CYP, the difference in size between the molecule size of IDM and the pore size of MPS_2_ was not more than 20 times. The pore size of MPS_2_ at 60 Å is 4–5 times larger than the size of IDM, thus MPS_2_ could inhibit the IDM molecules from forming a nucleus and suppress subsequent IDM crystallization. Moreover, the interaction between IDM and the silica surface could further stabilize the IDM amorphous within MPS_2_. The interaction between the carbonyl groups of IDM with silanol groups of SBA-15 through hydrogen bonding has been reported [38]. The experimental maximum loading amount of IDM was about 20%, which is lower than the theoretical value of MCM in MPS_2_ (26.75%). The loading efficiency of IDM incorporated into MPS_2_ was about 75%, which agrees with the previous study. Similar to RTV, although the interaction between IDM and silica surface was reported, some IDM was carried away from mesopores by the solvent in the drying process, and thus the loading amount of IDM was not completely 100%.

In this study, SAC, which was incorporated into MPS1, was evaluated as a comparison study due to its high recrystallization tendency (class I). The crystallization of SAC was observed in MPS_1_. The difference in size between the molecule size of SAC and the pore size of MPS_1_ was more than 20 times; thus, the recrystallization of SAC occurred in MPS_1_. Moreover, the weak interaction with the silica surface, the high mobility of SAC and its high recrystallization tendency, could further induce the formation of the critical nucleus crystal and subsequent recrystallization of SAC. The entire MPS_1_ could accommodate the molecules to be multilayers; thus, the maximum loading amount of SAC/MPS_1_ was very close to the theoretical calculation of PFC.

## 4. Materials and Methods

### 4.1. Materials

**Figure 10:**
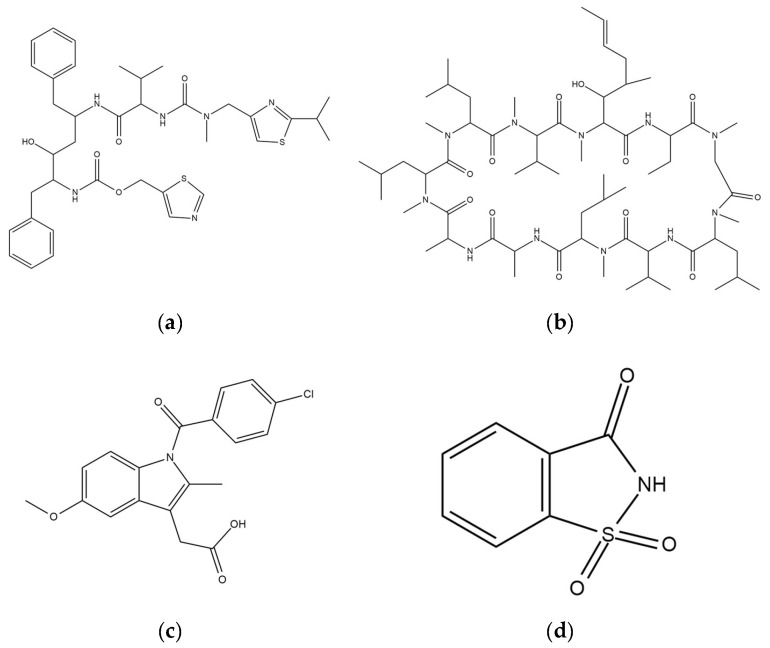
Chemical structures of (**a**) RTV, (**b**) CYP, (**c**) IDM and (**d**) SAC.

RTV (MW = 720.95 g/mol) was purchased from ChemShuttle (Hayward, Berkeley Heights, NJ, USA), while CYP (MW = 1202.61 g/mol), IDM (357.79) and SAC (MW = 183.18 g/mol) were purchased from FUJIFILM Wako Pure Chemical Corporation (Osaka, Japan). Their chemical structures are represented in Figure 10. Furthermore, MPS_1_ and MPS_2_ were kindly gifted from Taiyo Kagaku., Ltd (Mie, Japan). The pore volume, specific surface area and pore diameter of MPS_1_ were 0.92 cm^3^/g, 820 nm^2^/g and 8 nm, respectively, while for MPS_2_ they were 1.2 cm^3^/g, 550 nm^2^/g and 6 nm, respectively.

### 4.2. Preparation of Drug Loaded-MPS by the Solvent Evaporation Method

Each drug was dissolved in chloroform and then MPS was dispersed in the chloroform solution containing the drug with various weight ratios. The suspension was sonicated at 25 °C for 3 min, and evaporated using a rotary evaporator with a water bath for 30 min at 30 °C. The resulting powder was dried at 30 °C using a vacuum dryer for 48 h to obtain RTV/MPS_1_, CYP/MPS_1_, IDM/MPS_2_ and SAC/MPS_1_.

### 4.3. Preparation of Drug Loaded-MPS by Melt Method

The preparation of drug/MPS was conducted using a DSC with nitrogen purge gas and Freon intra cooling system. First, the physical mixtures of drug and MPS powder with various weight ratios were placed into a crimped aluminum DSC pan, heated above 10 °C of the drug’s melting point using a heating rate of 10 °C/min and held for 10 min. Afterwards, the samples were quenched until at −20 °C. Then, calorimetric analysis was conducted by heating the samples above the drug’s melting point using a heating rate of 10 °C/min.

### 4.4. MDSC Measurement

MDSC measurement was performed using a DSC-7000X instrument (Hitachi High-Tech Science Corporation; Tokyo, Japan). Approximately 5 mg of the sample was placed into a crimped aluminum DSC pan under an N_2_ purge at a 50 mL/min flow rate. Afterward, the samples were measured from 0 to 10 °C above the melting point of each drug at a heating rate of 2 °C/min with modulation of ±0.5 °C every 60 s.

### 4.5. Theoretical Calculation of the MCM and PFC

The theoretical monolayer coverage of each drug within MPS is calculated using the following Equation (1)

(1) $$X=\frac{\;SSA_{\mathrm{MPS}}\times 10^{20\;}\times MW_{\mathrm{drug}}\;\;}{S_{\mathrm{drug}}\times N_{A}}$$

where *X* is the capacity of each drug required for a monolayer coverage of MPS (g/g), *MW*_drug_ is the molecular weight of each drug (RTV = 720.95 g/mol, CYP = 1202.61 g/mol), IDM = 357.79 g/mol and SAC = 183.18 g/mol), *S*_drug_ is the molecular contact surface area of each drug, *SSA*_MPS_ is the specific surface area of MPS (MPS_1_ = 820 m^2^/g and MPS_2_ = 550 m^2^/g) and $N_{A}$ is the number of Avogadro (6.022 × 10^23^ mol^−1^).

The maximum theoretical load of each drug inside the pores of MPS, referred to as pore-filling capacity (PFC), was determined based on pore volume by utilizing the density of each drug, according to the following Equation (2)

(2) $$Y=\frac{\;V_{\mathrm{MPS}}\times \rho_{\mathrm{drug}}\;\;}{1+V_{\mathrm{MPS}}\times \rho_{\mathrm{drug}}}$$

where *Y* is the maximum theoretical load of each drug in the monolayer and the excess drug confined by the pores of MPS (g/g). V_MPS_ is the pore volume of the MPS (MPS_1_ = 0.92 cm^3^/g and MPS_2_ = 1.2cm^3^/g), while ρ_drug_ is the molecular density of each drug [15,20,49,50].

### 4.6. PXRD Measurement

The PXRD measurement was performed by a Miniflex II (Rigaku Co., Ltd, Tokyo, Japan) with the following conditions: target, Cu; filter, Ni; voltage, 30 kV; current, 15 mA; scanning rate, 4°/min and scanning angle of 2θ = 3–40°.

### 4.7. Solid-State ^13^C NMR Measurement

Solid-state ^13^C NMR was conducted by a JNM-ECX-400 NMR system (9.4 T; JEOL Resonance Inc., Tokyo, Japan) with a JEOL 4 mm HXMAS probe and the samples were measured using the CP/MAS of spinning sidebands experiments under the following conditions: spinning rate, 5 kHz; contact time, 2 ms; scans, 55,000; relaxation delay, 5 s.

## 5. Conclusions

In this study, the characterization of drugs with low recrystallization tendency within MPS, experimental determination of maximum drug loading and its relevance to theoretical value, referred to as theoretical monolayer coverage of drugs within MPS pore-filling capacity, was elucidated. The drugs were selected with good glass formers representing a group of substances with rather small (IDM), medium (RTV) and fairly large (CYP) molecular sizes, while SAC, which has small molecular size and poor glass formers, was used as a comparison study. The plots of drug concentration against the Δ*Cp* of amorphous drug and the absence of *T*_g_ in the MDSC curve could be used to determine the maximum loading amount within MPS of drugs that are good glass formers, while the lack of *T*_m_ could be used for the drugs that are poor glass formers and/or recrystallize quickly in the heating step during the DSC run. The theoretical value of drug-loaded MPS either from monolayer covering the surface of mesoporous or from the pore filling capacity was not continuously relevant with the experimental maximum drug loading amounts. The characterization of drugs within MPS, such as molecular size and interaction of drug-silica surface, could affect the loading efficiency of drugs within MPS that influence its relevance with the theoretical value of drugs within MPS. This study provided fundamental insight into the formulation of amorphous drugs incorporated into mesoporous silica, specifically in determining the maximum loading amount of drugs to achieve high doses in the MPS system.

## Data Availability

The original contributions presented in the study are publicly available.

## Supplementary Materials

The following are available online at https://www.mdpi.com/article/10.3390/ph15010093/s1, Figure S1: FT-IR spectrum of MS1 and MS2, in the OH stretch region, Figure S2: ^29^Si NMR spectra of MS_1_ and MS_2_, Figure S3: Nonreversible signal of (a) RTV EVPs, RTV/MPS_1_ = (b) 8:2, (c) 7:3, (d) 6:4, (e) 5:5, (f) 4:6, (g) 3:7, and (h) MPS_1_, Figure S4: Nonreversible signal of (a) CYP amorphous, CYP/MPS_1_ = (b) 7:3, (c) 5:5, and (d) 3:7, Figure S5: Nonreversible signal of (a) γ-IDM crystal, IDM/MPS_2_ = (b) 4:6, (c) 3:7, (d) 2:8, (e)1.5:8.5, and (f) MPS_2_, Figure S6: Nonreversible signal of (a) SAC crystal, evaporated sample of SAC/MPS_1_ = (b) 4:6, (c) 3:7, (d) 2:8, (e) 1.5:8.5, and (f) 1:9.
