# Supplementary material for: Characterization of Drugs with Good Glass Formers in Loaded-Mesoporous Silica and Its Theoretical Value Relevance with Mesopores Surface and Pore-Filling Capacity
Source: Pharmaceuticals (Basel). 2022 Jan 13;15(1):93. doi: 10.3390/ph15010093 (PMC8778383; doi:10.3390/ph15010093)
Supplement: Supplementary file 1 [file pharmaceuticals-15-00093-s001.zip › pharmaceuticals-1495312-supplementary.pdf]

Supplementary Material

FT-IR spectrum of MPS_1 and MPS_2.
Method :
FT-IR measurements were conducted on FT-IR spectrometer (Bruker Optik GmbH, Ettlingen, Germany), using KBr tablet method. FT-IR spectra were obtained in the scan range of 400–4000 cm^{-1} at a resolution of 4 cm^{-1}.

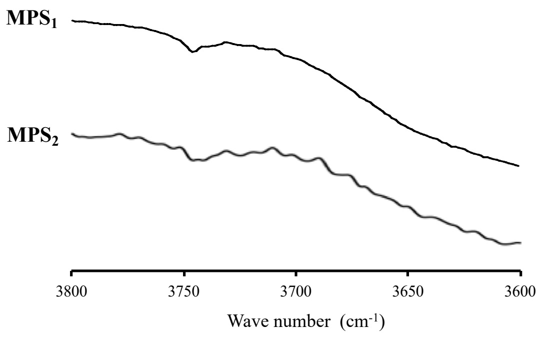

Figure S1. FT-IR spectrum of MS1 and MS2, in the OH stretch region.

Previous studies reported that MPS possesses a characteristic signal at around 3749 cm^{-1} assigned to the stretching vibrations of isolated (i.e. non-hydrogen bonded) silanol groups [31,48]. MS_1 and MS_2 exhibited a characteristic signal at 3750 cm^{-1} attributed to the stretching vibrations of isolated (i.e. non-hydrogen bonded) silanol groups. This indicated that on the surface of MPS could be only OH groups.

^{29}Si NMR spectra of MS_1 and MS_2

Method :

Solid-state ^{29}Si NMR was performed using a JNM-ECX-400 NMR system (9.4 T; JEOL Resonance Inc., Tokyo, Japan) equipped with a JEOL 4 mm HXMAS probe. A total of 10,000 scans were accumulated under the following conditions: spinning rate, 5 kHz; contact time, 2 ms. The samples were packed in 4 mm Zirconia rotors; while Tetramethylsilane was used as shift reference.

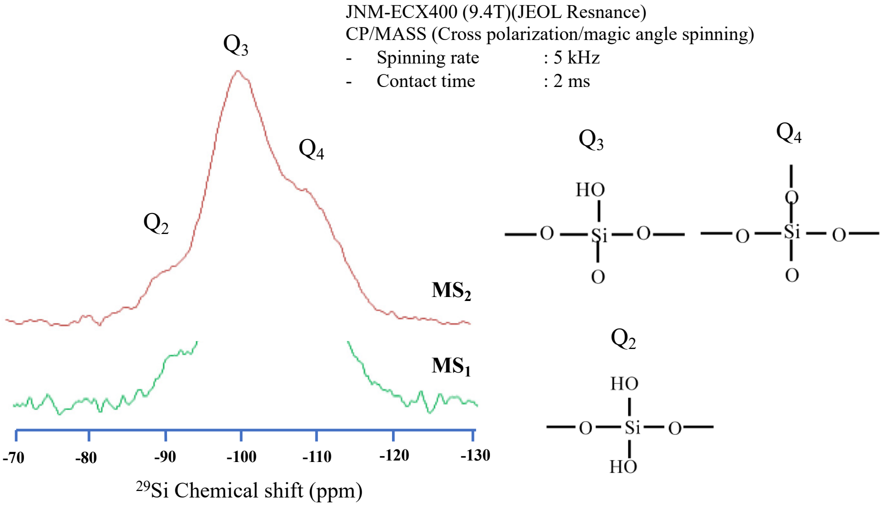

Figure S2. ^{29}Si NMR spectra of MS_1 and MS_2.

The Q2 and Q3, which was attributed to the silanol groups, was observed in both MS_1 and MS_2. This indicated that OH groups was observed on the silica surface of MS_1 and MS_2.

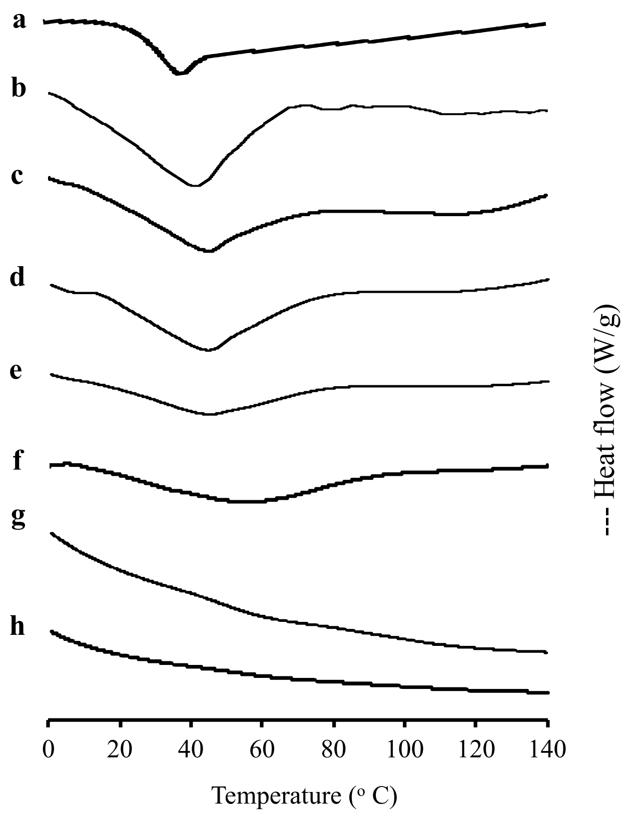

Figure S3. Nonreversible signal of (a) RTV EVPs, RTV/MPS1 = (b) 8:2, (c) 7:3, (d) 6:4, (e) 5:5, (f) 4:6, (g) 3:7, and (h) MPS1.

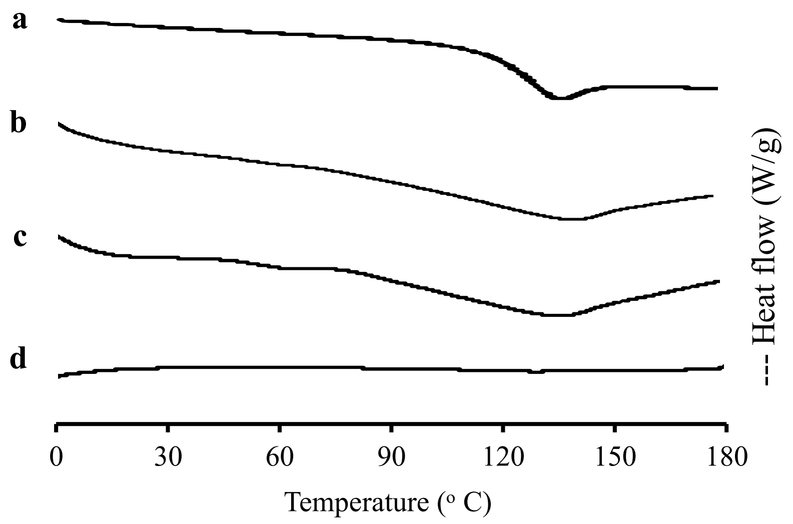

Figure S4. Nonreversible signal of (a) CYP amorphous, CYP/MPS1 = (b) 7:3, (c) 5:5, and (d) 3:7.

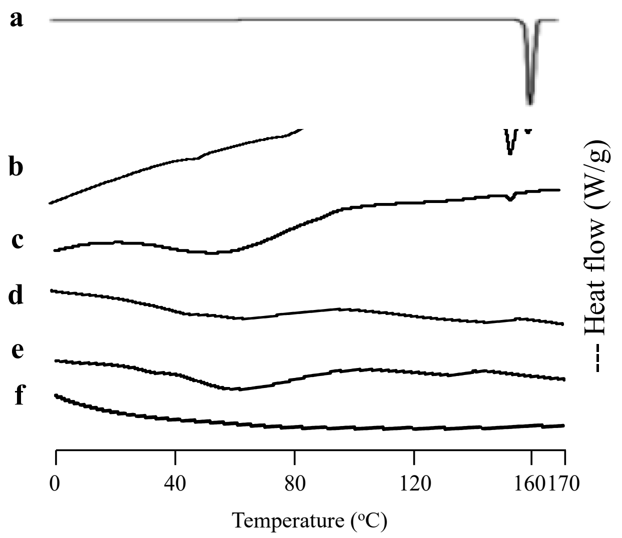

Figure S5. Nonreversible signal of (a) γ-IDM crystal, IDM/MPS_2 = (b) 4:6, (c) 3:7, (d) 2:8, (e)1.5:8.5, and (f) MPS_2.

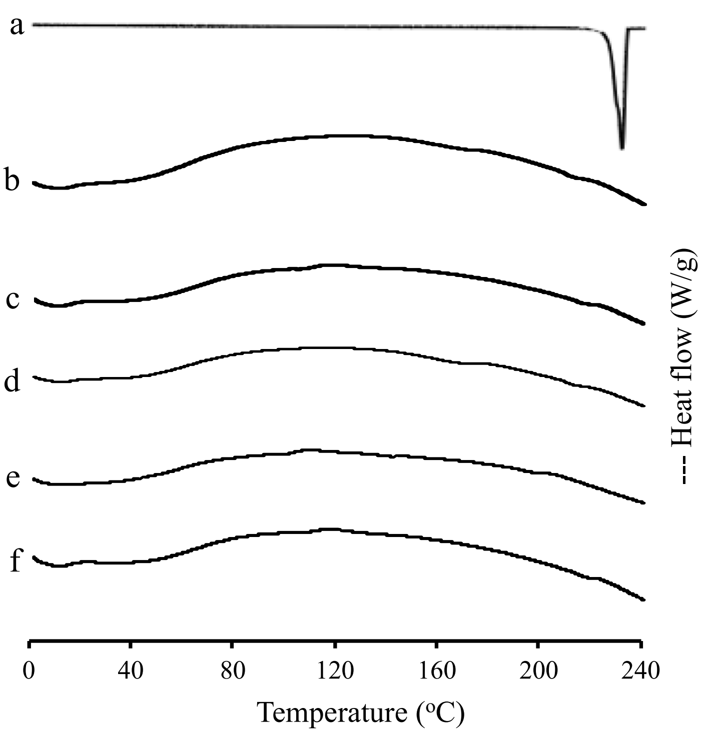

Figure S6. Nonreversible signal of (a) SAC crystal, evaporated sample of SAC/MPS1 = (b) 4:6, (c) 3:7, (d) 2:8, (e) 1.5:8.5, and (f) 1:9.
